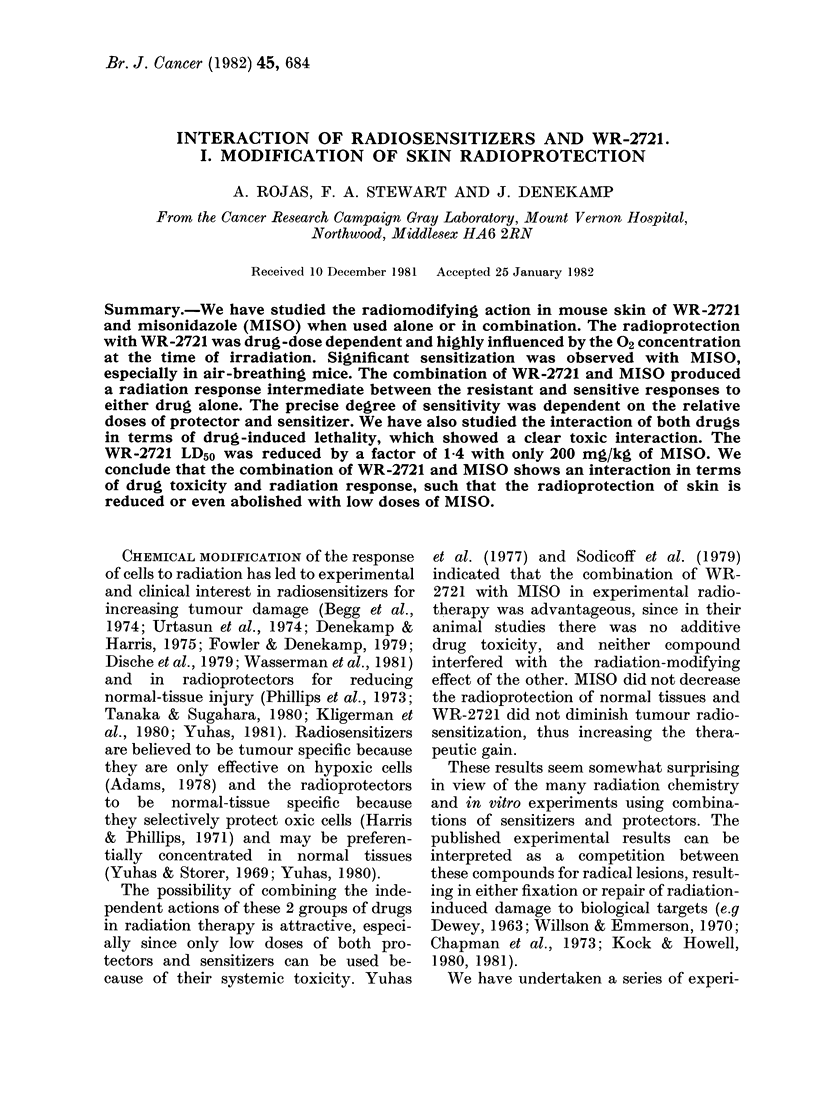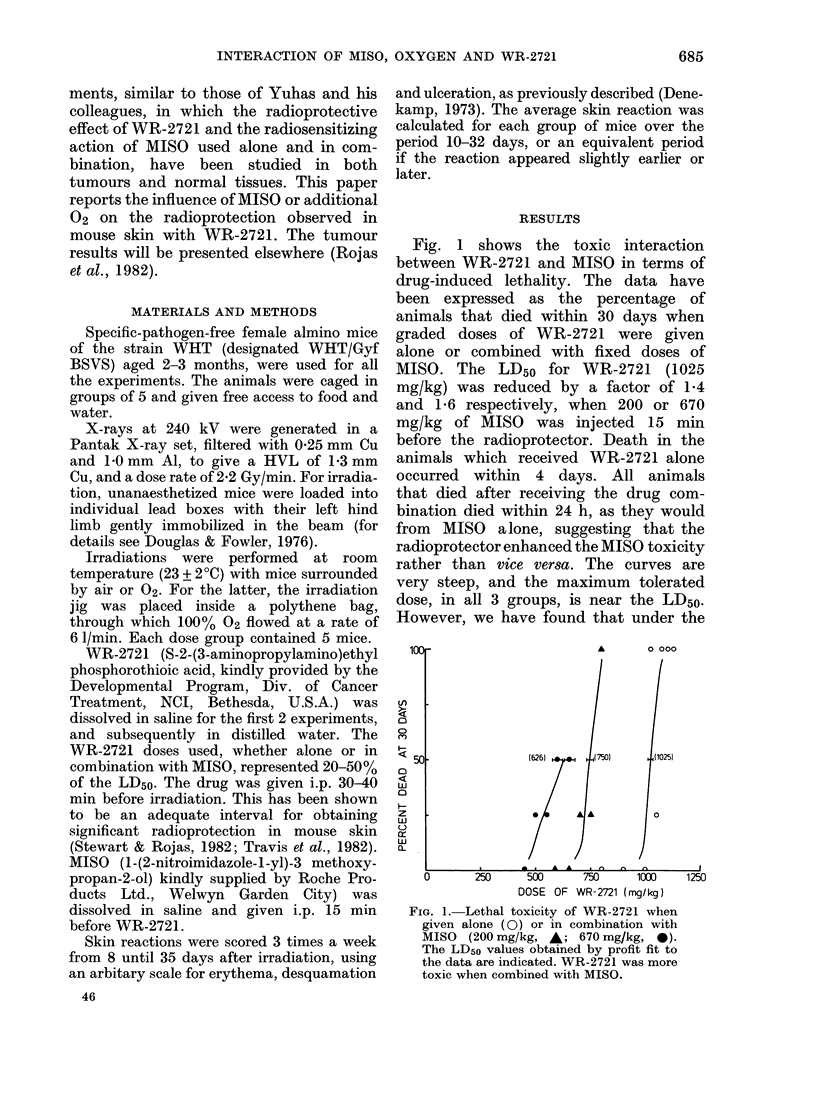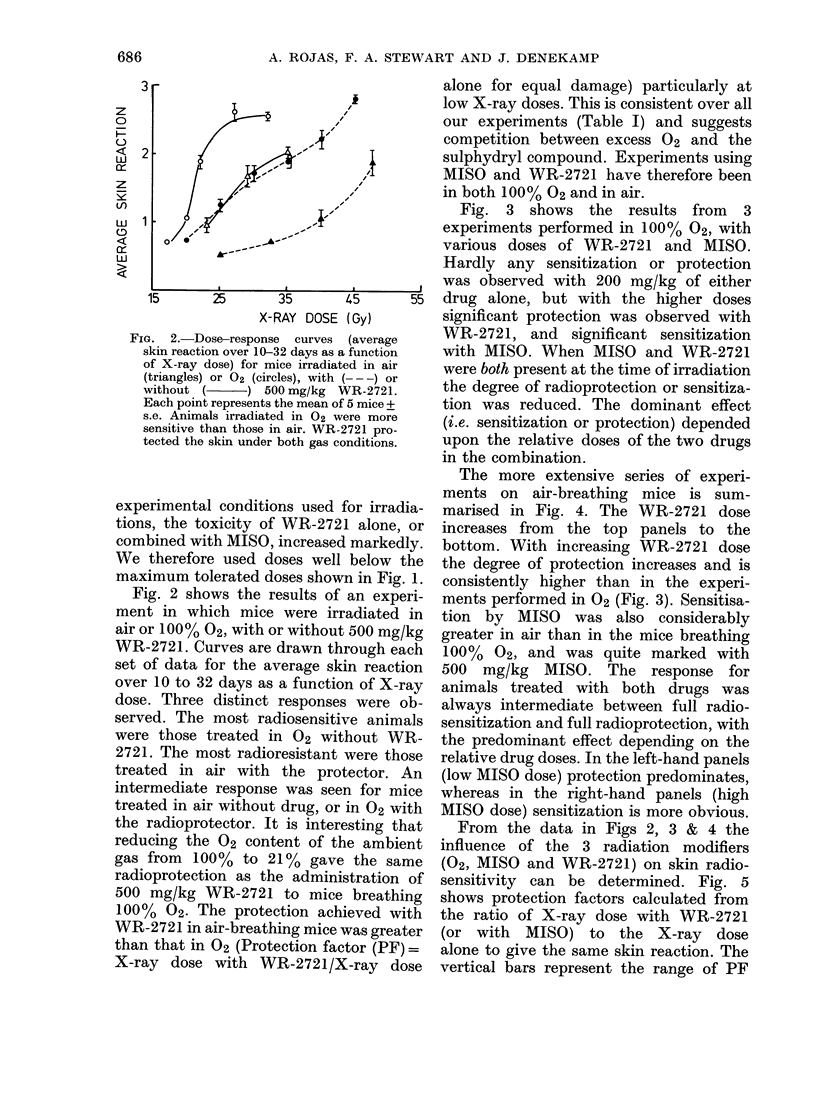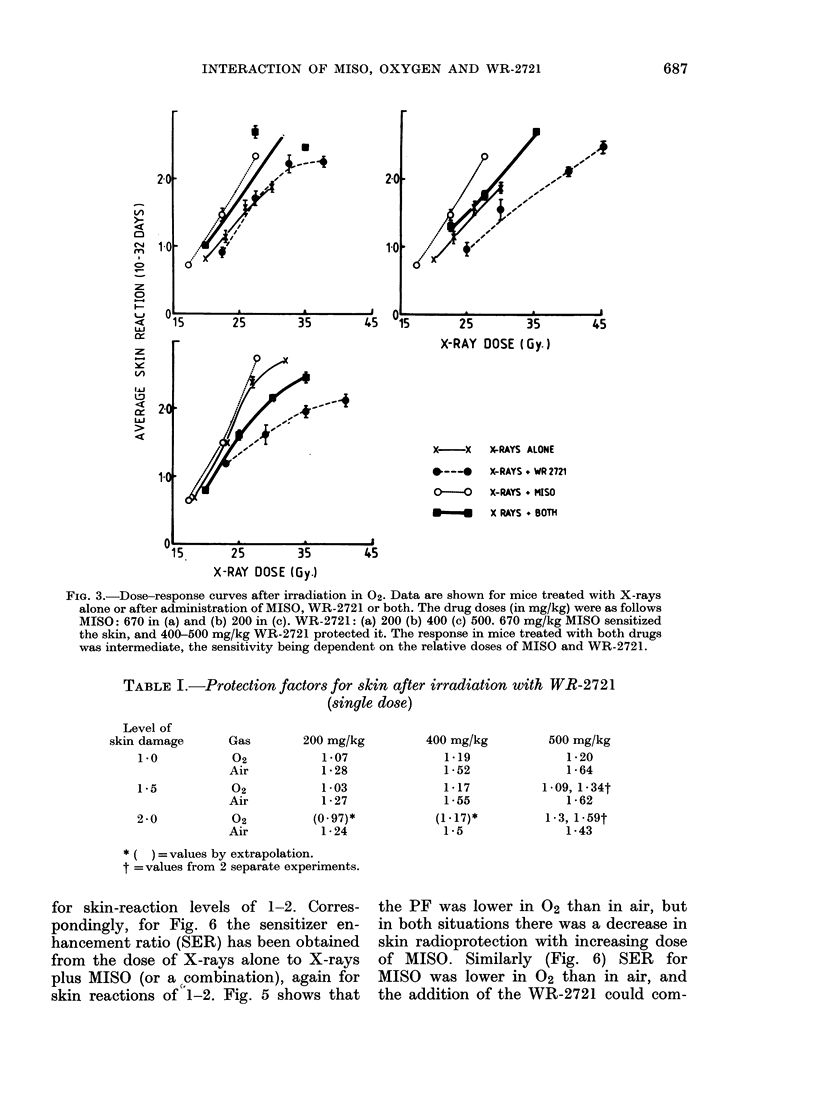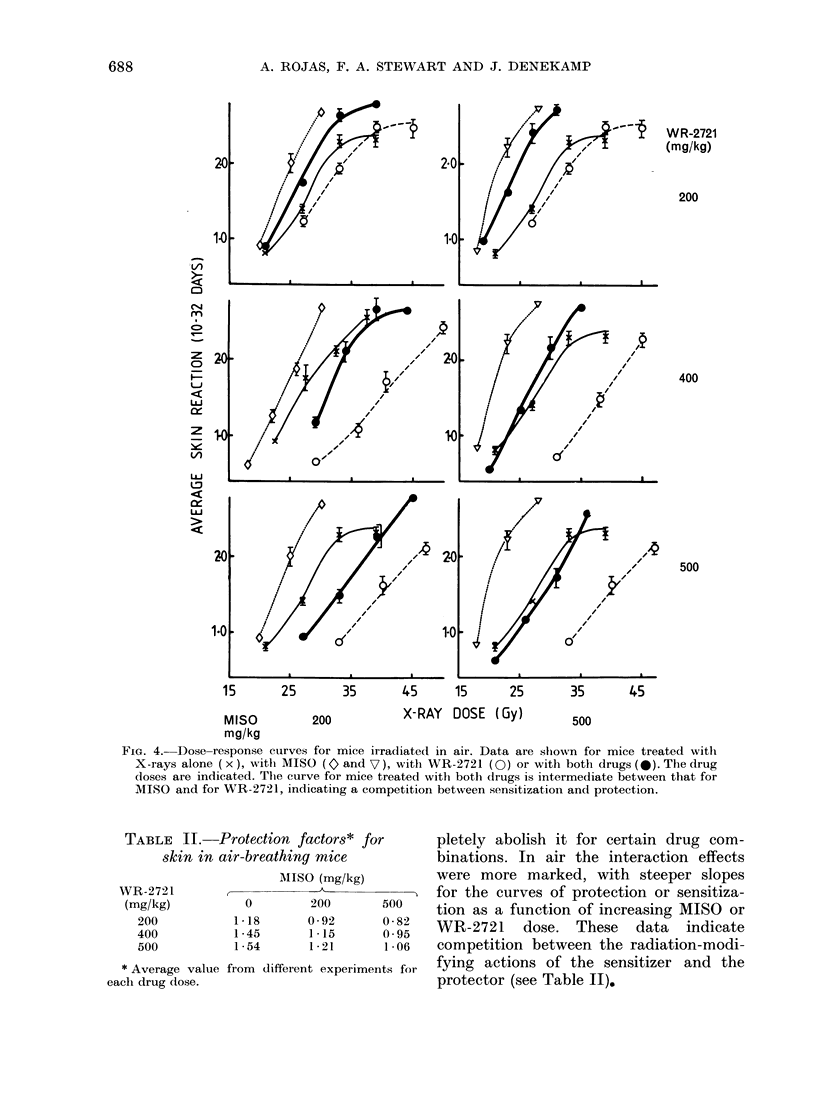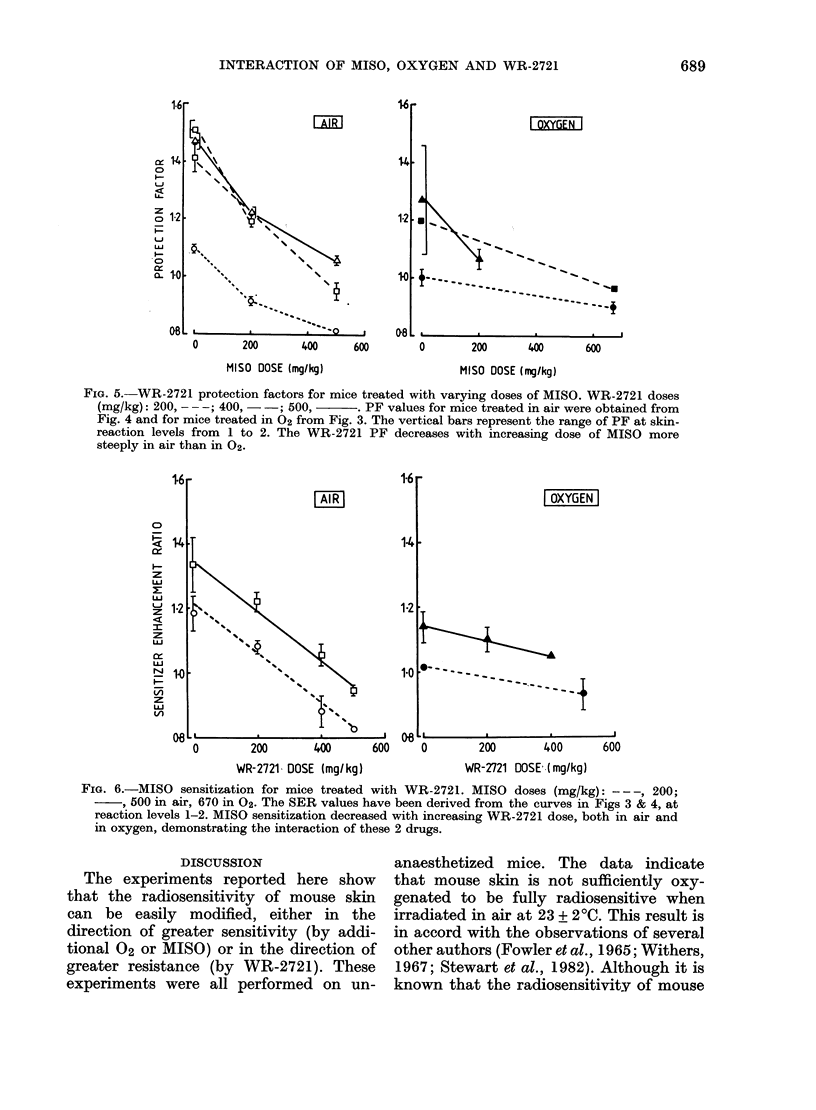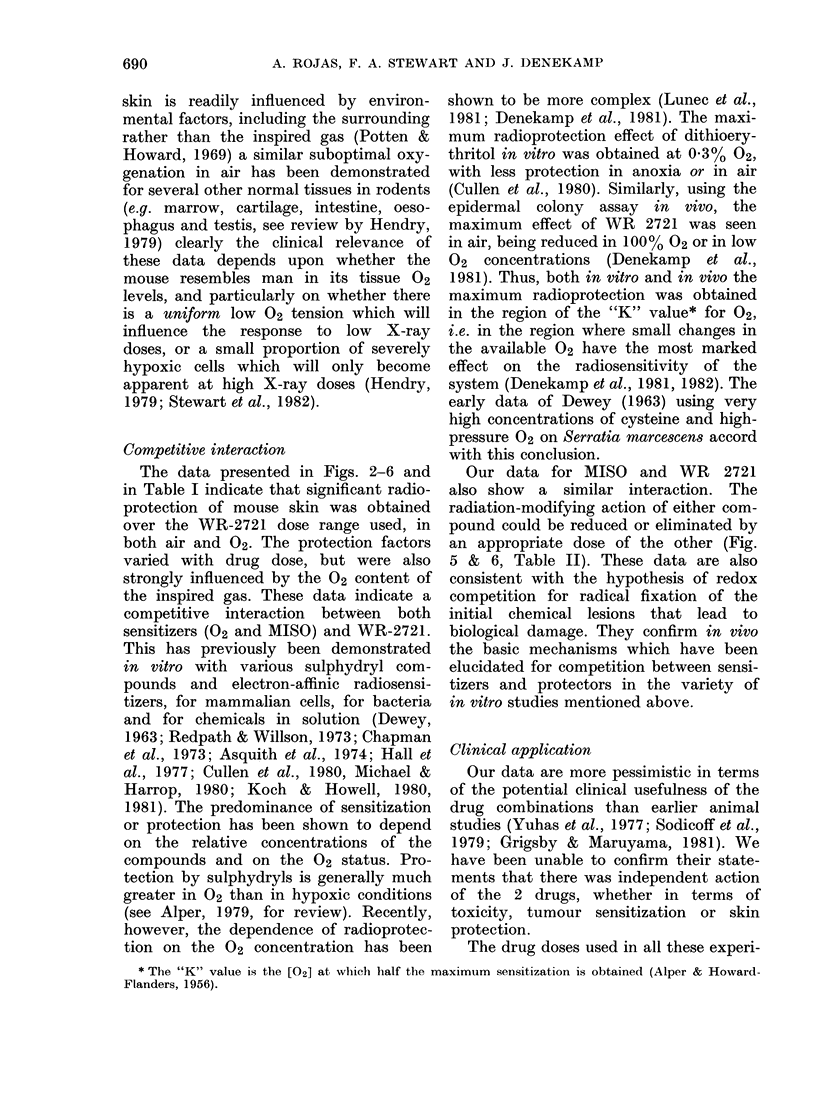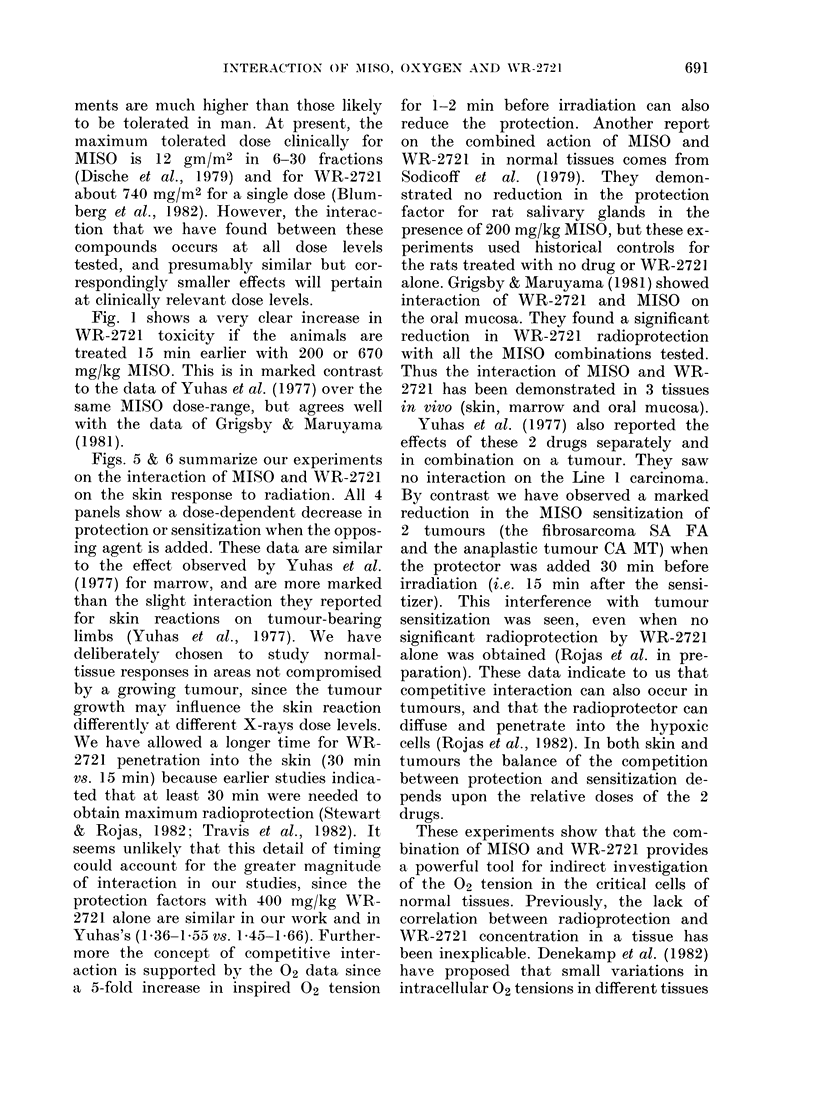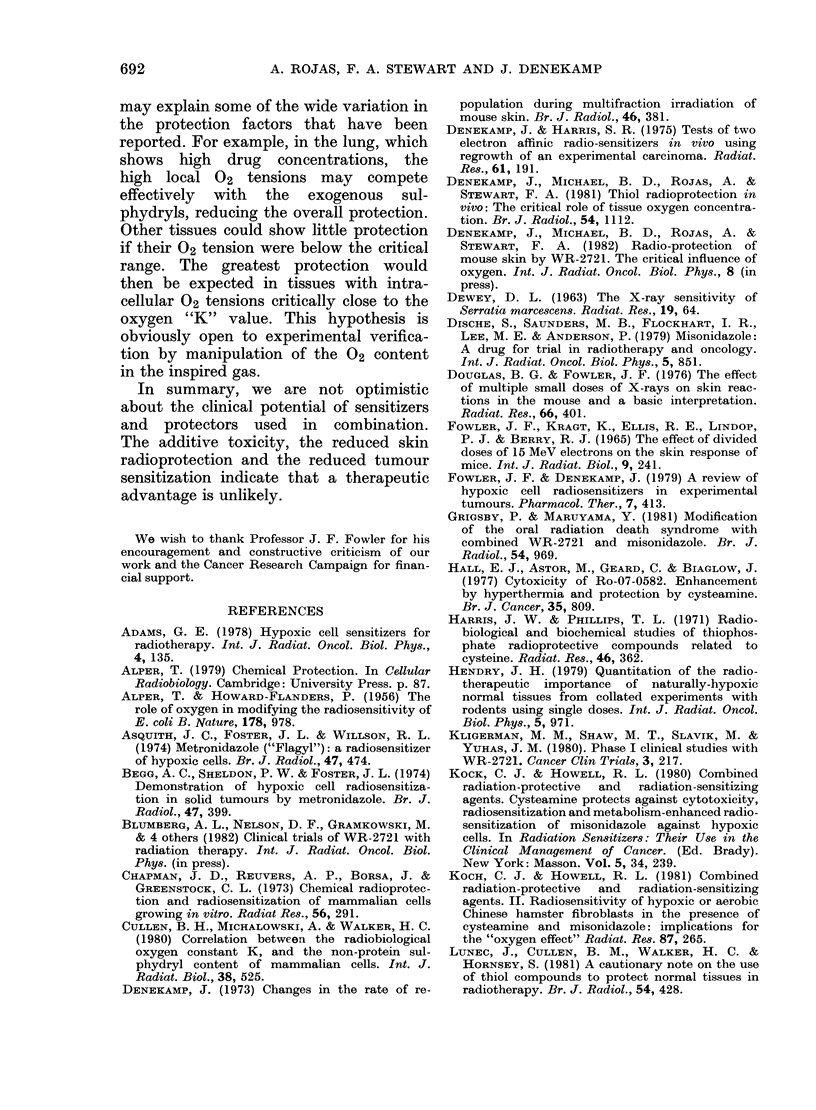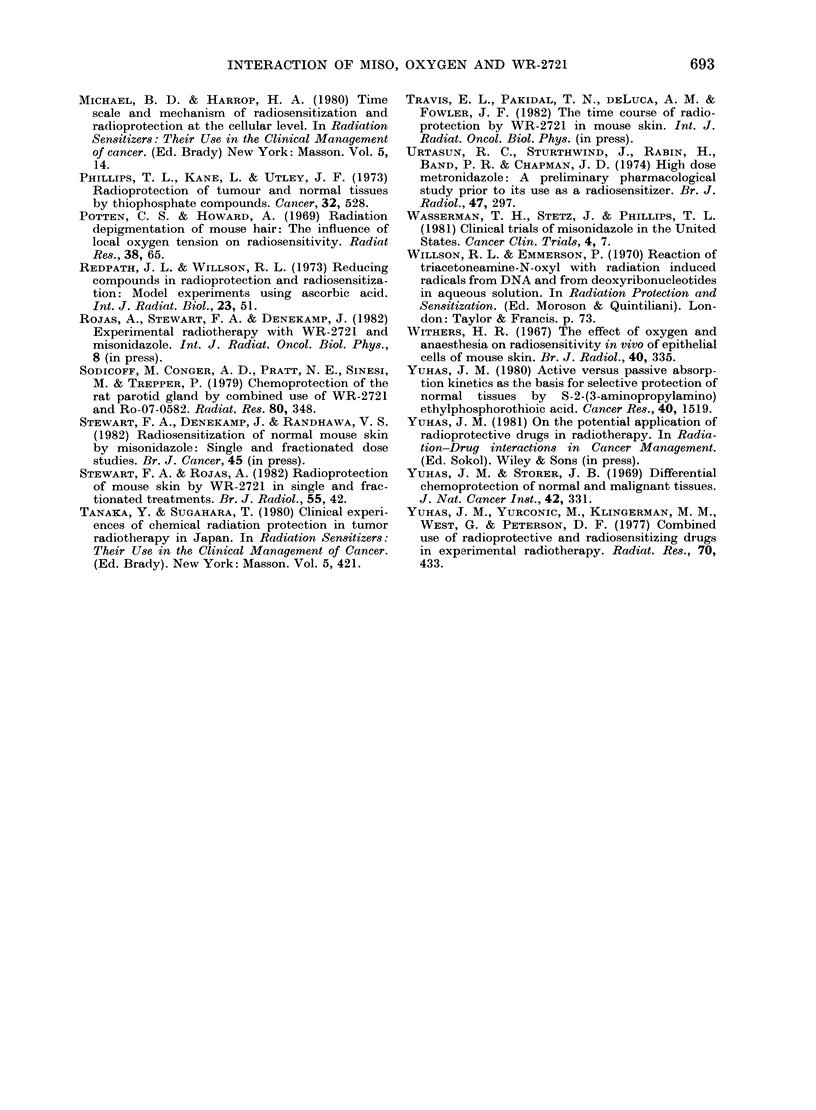# Interaction of radiosensitizers and WR-2721. I. Modification of skin radioprotection.

**DOI:** 10.1038/bjc.1982.109

**Published:** 1982-05

**Authors:** A. Rojas, F. A. Stewart, J. Denekamp

## Abstract

We have studied the radiomodifying action in mouse skin of WR-2721 and misonidazole (MISO) when used alone or in combination. The radioprotection with WR-2721 was drug-dose dependent and highly influenced by the O2 concentration at the time of irradiation. Significant sensitizaton was observed with MISO, especially in air-breathing mice. The combination of WR-2721 and MISO produced a radiation response intermediate between the resistant and sensitive responses to either drug alone. The precise degree of sensitivity was dependent on the relative doses of protector and sensitizer. We have also studied the interaction of both drugs in terms of drug-induced lethality, which showed a clear toxic interaction. The WR-2721 LD50 was reduced by a factor of 1.4 with only 200 mg/kg of MISO. We conclude that the combination of WR-2721 and MISO shows an interaction in terms of drug toxicity and radiation response, such that the radioprotection of skin is reduced or even abolished with low doses of MISO.


*Br. J. Cancer* (1982) **45**, 684

### INTERACTION OF RADIOSENSITIZERS AND WR-2721. I. MODIFICATION OF SKIN RADIOPROTECTION

A. ROJAS, F. A. STEWART AND J. DENEKAMP

*From the Cancer Research Campaign Gray Laboratory, Mount Vernon Hospital, Northwood, Middlesex HA6 2RN*

Received 10 December 1981 Accepted 25 January 1982

**Summary.—We have studied the radiomodifying action in mouse skin of WR-2721 and misonidazole (MISO) when used alone or in combination. The radioprotection with WR-2721 was drug-dose dependent and highly influenced by the $O_2$ concentration at the time of irradiation. Significant sensitization was observed with MISO, especially in air-breathing mice. The combination of WR-2721 and MISO produced a radiation response intermediate between the resistant and sensitive responses to either drug alone. The precise degree of sensitivity was dependent on the relative doses of protector and sensitizer. We have also studied the interaction of both drugs in terms of drug-induced lethality, which showed a clear toxic interaction. The WR-2721 $LD_{50}$ was reduced by a factor of 1·4 with only 200 mg/kg of MISO. We conclude that the combination of WR-2721 and MISO shows an interaction in terms of drug toxicity and radiation response, such that the radioprotection of skin is reduced or even abolished with low doses of MISO.**

Chemical modification of the response of cells to radiation has led to experimental and clinical interest in radiosensitizers for increasing tumour damage (Begg *et al.*, 1974; Urtasun *et al.*, 1974; Denekamp & Harris, 1975; Fowler & Denekamp, 1979; Dische *et al.*, 1979; Wasserman *et al.*, 1981) and in radioprotectors for reducing normal-tissue injury (Phillips *et al.*, 1973; Tanaka & Sugahara, 1980; Kligerman *et al.*, 1980; Yuhas, 1981). Radiosensitizers are believed to be tumour specific because they are only effective on hypoxic cells (Adams, 1978) and the radioprotectors to be normal-tissue specific because they selectively protect oxic cells (Harris & Phillips, 1971) and may be preferentially concentrated in normal tissues (Yuhas & Storer, 1969; Yuhas, 1980).

The possibility of combining the independent actions of these 2 groups of drugs in radiation therapy is attractive, especially since only low doses of both protectors and sensitizers can be used because of their systemic toxicity. Yuhas *et al.* (1977) and Sodicoff *et al.* (1979) indicated that the combination of WR-2721 with MISO in experimental radiotherapy was advantageous, since in their animal studies there was no additive drug toxicity, and neither compound interfered with the radiation-modifying effect of the other. MISO did not decrease the radioprotection of normal tissues and WR-2721 did not diminish tumour radiosensitization, thus increasing the therapeutic gain.

These results seem somewhat surprising in view of the many radiation chemistry and *in vitro* experiments using combinations of sensitizers and protectors. The published experimental results can be interpreted as a competition between these compounds for radical lesions, resulting in either fixation or repair of radiation-induced damage to biological targets (*e.g* Dewey, 1963; Willson & Emmerson, 1970; Chapman *et al.*, 1973; Kock & Howell, 1980, 1981).

We have undertaken a series of experiments, similar to those of Yuhas and his colleagues, in which the radioprotective effect of WR-2721 and the radiosensitizing action of MISO used alone and in combination, have been studied in both tumours and normal tissues. This paper reports the influence of MISO or additional $O_2$ on the radioprotection observed in mouse skin with WR-2721. The tumour results will be presented elsewhere (Rojas *et al.*, 1982).

### MATERIALS AND METHODS

Specific-pathogen-free female almino mice of the strain WHT (designated WHT/Gyf BSVS) aged 2–3 months, were used for all the experiments. The animals were caged in groups of 5 and given free access to food and water.

X-rays at 240 kV were generated in a Pantak X-ray set, filtered with 0·25 mm Cu and 1·0 mm Al, to give a HVL of 1·3 mm Cu, and a dose rate of 2·2 Gy/min. For irradiation, unanaesthetized mice were loaded into individual lead boxes with their left hind limb gently immobilized in the beam (for details see Douglas & Fowler, 1976).

Irradiations were performed at room temperature (23 ± 2°C) with mice surrounded by air or $O_2$. For the latter, the irradiation jig was placed inside a polythene bag, through which 100% $O_2$ flowed at a rate of 6 l/min. Each dose group contained 5 mice.

WR-2721 (S-2-(3-aminopropylamino)ethyl phosphorothioic acid, kindly provided by the Developmental Program, Div. of Cancer Treatment, NCI, Bethesda, U.S.A.) was dissolved in saline for the first 2 experiments, and subsequently in distilled water. The WR-2721 doses used, whether alone or in combination with MISO, represented 20–50% of the $LD_{50}$. The drug was given i.p. 30–40 min before irradiation. This has been shown to be an adequate interval for obtaining significant radioprotection in mouse skin (Stewart & Rojas, 1982; Travis *et al.*, 1982). MISO (1-(2-nitroimidazole-1-yl)-3 methoxy-propan-2-ol) kindly supplied by Roche Products Ltd., Welwyn Garden City) was dissolved in saline and given i.p. 15 min before WR-2721.

Skin reactions were scored 3 times a week from 8 until 35 days after irradiation, using an arbitary scale for erythema, desquamation and ulceration, as previously described (Denekamp, 1973). The average skin reaction was calculated for each group of mice over the period 10–32 days, or an equivalent period if the reaction appeared slightly earlier or later.

### RESULTS

Fig. 1 shows the toxic interaction between WR-2721 and MISO in terms of drug-induced lethality. The data have been expressed as the percentage of animals that died within 30 days when graded doses of WR-2721 were given alone or combined with fixed doses of MISO. The $LD_{50}$ for WR-2721 (1025 mg/kg) was reduced by a factor of 1·4 and 1·6 respectively, when 200 or 670 mg/kg of MISO was injected 15 min before the radioprotector. Death in the animals which received WR-2721 alone occurred within 4 days. All animals that died after receiving the drug combination died within 24 h, as they would from MISO alone, suggesting that the radioprotector enhanced the MISO toxicity rather than *vice versa*. The curves are very steep, and the maximum tolerated dose, in all 3 groups, is near the $LD_{50}$. However, we have found that under the

Fig. 1.—Lethal toxicity of WR-2721 when given alone (○) or in combination with MISO (200 mg/kg, ▲; 670 mg/kg, ●). The $LD_{50}$ values obtained by profit fit to the data are indicated. WR-2721 was more toxic when combined with MISO.

FIG. 2.—Dose–response curves (average skin reaction over 10–32 days as a function of X-ray dose) for mice irradiated in air (triangles) or $O_2$ (circles), with (– – –) or without (———) 500 mg/kg WR-2721. Each point represents the mean of 5 mice ± s.e. Animals irradiated in $O_2$ were more sensitive than those in air. WR-2721 protected the skin under both gas conditions.

experimental conditions used for irradiations, the toxicity of WR-2721 alone, or combined with MISO, increased markedly. We therefore used doses well below the maximum tolerated doses shown in Fig. 1.

Fig. 2 shows the results of an experiment in which mice were irradiated in air or 100% $O_2$, with or without 500 mg/kg WR-2721. Curves are drawn through each set of data for the average skin reaction over 10 to 32 days as a function of X-ray dose. Three distinct responses were observed. The most radiosensitive animals were those treated in $O_2$ without WR-2721. The most radioresistant were those treated in air with the protector. An intermediate response was seen for mice treated in air without drug, or in $O_2$ with the radioprotector. It is interesting that reducing the $O_2$ content of the ambient gas from 100% to 21% gave the same radioprotection as the administration of 500 mg/kg WR-2721 to mice breathing 100% $O_2$. The protection achieved with WR-2721 in air-breathing mice was greater than that in $O_2$ (Protection factor (PF) = X-ray dose with WR-2721/X-ray dose alone for equal damage) particularly at low X-ray doses. This is consistent over all our experiments (Table I) and suggests competition between excess $O_2$ and the sulphydryl compound. Experiments using MISO and WR-2721 have therefore been in both 100% $O_2$ and in air.

Fig. 3 shows the results from 3 experiments performed in 100% $O_2$, with various doses of WR-2721 and MISO. Hardly any sensitization or protection was observed with 200 mg/kg of either drug alone, but with the higher doses significant protection was observed with WR-2721, and significant sensitization with MISO. When MISO and WR-2721 were *both* present at the time of irradiation the degree of radioprotection or sensitization was reduced. The dominant effect (*i.e.* sensitization or protection) depended upon the relative doses of the two drugs in the combination.

The more extensive series of experiments on air-breathing mice is summarised in Fig. 4. The WR-2721 dose increases from the top panels to the bottom. With increasing WR-2721 dose the degree of protection increases and is consistently higher than in the experiments performed in $O_2$ (Fig. 3). Sensitisation by MISO was also considerably greater in air than in the mice breathing 100% $O_2$, and was quite marked with 500 mg/kg MISO. The response for animals treated with both drugs was always intermediate between full radiosensitization and full radioprotection, with the predominant effect depending on the relative drug doses. In the left-hand panels (low MISO dose) protection predominates, whereas in the right-hand panels (high MISO dose) sensitization is more obvious.

From the data in Figs 2, 3 & 4 the influence of the 3 radiation modifiers ($O_2$, MISO and WR-2721) on skin radiosensitivity can be determined. Fig. 5 shows protection factors calculated from the ratio of X-ray dose with WR-2721 (or with MISO) to the X-ray dose alone to give the same skin reaction. The vertical bars represent the range of PF

FIG. 3.—Dose–response curves after irradiation in $O_2$. Data are shown for mice treated with X-rays alone or after administration of MISO, WR-2721 or both. The drug doses (in mg/kg) were as follows MISO: 670 in (a) and (b) 200 in (c). WR-2721: (a) 200 (b) 400 (c) 500. 670 mg/kg MISO sensitized the skin, and 400–500 mg/kg WR-2721 protected it. The response in mice treated with both drugs was intermediate, the sensitivity being dependent on the relative doses of MISO and WR-2721.

TABLE I.—*Protection factors for skin after irradiation with WR-2721 (single dose)*

| Level of skin damage | Gas | 200 mg/kg | 400 mg/kg | 500 mg/kg |
|---|---|---|---|---|
| 1·0 | $O_2$ | 1·07 | 1·19 | 1·20 |
| | Air | 1·28 | 1·52 | 1·64 |
| 1·5 | $O_2$ | 1·03 | 1·17 | 1·09, 1·34† |
| | Air | 1·27 | 1·55 | 1·62 |
| 2·0 | $O_2$ | (0·97)* | (1·17)* | 1·3, 1·59† |
| | Air | 1·24 | 1·5 | 1·43 |

* ( ) = values by extrapolation.
† = values from 2 separate experiments.

for skin-reaction levels of 1–2. Correspondingly, for Fig. 6 the sensitizer enhancement ratio (SER) has been obtained from the dose of X-rays alone to X-rays plus MISO (or a combination), again for skin reactions of 1–2. Fig. 5 shows that the PF was lower in $O_2$ than in air, but in both situations there was a decrease in skin radioprotection with increasing dose of MISO. Similarly (Fig. 6) SER for MISO was lower in $O_2$ than in air, and the addition of the WR-2721 could com-

FIG. 4.—Dose–response curves for mice irradiated in air. Data are shown for mice treated with X-rays alone (×), with MISO (◇ and ▽), with WR-2721 (○) or with both drugs (●). The drug doses are indicated. The curve for mice treated with both drugs is intermediate between that for MISO and for WR-2721, indicating a competition between sensitization and protection.

TABLE II.—*Protection factors* for skin in air-breathing mice*

| WR-2721 (mg/kg) | MISO (mg/kg) 0 | 200 | 500 |
|---|---|---|---|
| 200 | 1·18 | 0·92 | 0·82 |
| 400 | 1·45 | 1·15 | 0·95 |
| 500 | 1·54 | 1·21 | 1·06 |

* Average value from different experiments for each drug dose.

pletely abolish it for certain drug combinations. In air the interaction effects were more marked, with steeper slopes for the curves of protection or sensitization as a function of increasing MISO or WR-2721 dose. These data indicate competition between the radiation-modifying actions of the sensitizer and the protector (see Table II).

FIG. 5.—WR-2721 protection factors for mice treated with varying doses of MISO. WR-2721 doses (mg/kg): 200, - - -; 400, — —; 500, ———. PF values for mice treated in air were obtained from Fig. 4 and for mice treated in $O_2$ from Fig. 3. The vertical bars represent the range of PF at skin-reaction levels from 1 to 2. The WR-2721 PF decreases with increasing dose of MISO more steeply in air than in $O_2$.

FIG. 6.—MISO sensitization for mice treated with WR-2721. MISO doses (mg/kg): - - -, 200; ——, 500 in air, 670 in $O_2$. The SER values have been derived from the curves in Figs 3 & 4, at reaction levels 1–2. MISO sensitization decreased with increasing WR-2721 dose, both in air and in oxygen, demonstrating the interaction of these 2 drugs.

### DISCUSSION

The experiments reported here show that the radiosensitivity of mouse skin can be easily modified, either in the direction of greater sensitivity (by additional $O_2$ or MISO) or in the direction of greater resistance (by WR-2721). These experiments were all performed on unanaesthetized mice. The data indicate that mouse skin is not sufficiently oxygenated to be fully radiosensitive when irradiated in air at 23 ± 2°C. This result is in accord with the observations of several other authors (Fowler *et al.*, 1965; Withers, 1967; Stewart *et al.*, 1982). Although it is known that the radiosensitivity of mouse skin is readily influenced by environmental factors, including the surrounding rather than the inspired gas (Potten & Howard, 1969) a similar suboptimal oxygenation in air has been demonstrated for several other normal tissues in rodents (*e.g.* marrow, cartilage, intestine, oesophagus and testis, see review by Hendry, 1979) clearly the clinical relevance of these data depends upon whether the mouse resembles man in its tissue $O_2$ levels, and particularly on whether there is a *uniform* low $O_2$ tension which will influence the response to low X-ray doses, or a small proportion of severely hypoxic cells which will only become apparent at high X-ray doses (Hendry, 1979; Stewart *et al.*, 1982).

### *Competitive interaction*

The data presented in Figs. 2–6 and in Table I indicate that significant radioprotection of mouse skin was obtained over the WR-2721 dose range used, in both air and $O_2$. The protection factors varied with drug dose, but were also strongly influenced by the $O_2$ content of the inspired gas. These data indicate a competitive interaction between both sensitizers ($O_2$ and MISO) and WR-2721. This has previously been demonstrated *in vitro* with various sulphydryl compounds and electron-affinic radiosensitizers, for mammalian cells, for bacteria and for chemicals in solution (Dewey, 1963; Redpath & Willson, 1973; Chapman *et al.*, 1973; Asquith *et al.*, 1974; Hall *et al.*, 1977; Cullen *et al.*, 1980, Michael & Harrop, 1980; Koch & Howell, 1980, 1981). The predominance of sensitization or protection has been shown to depend on the relative concentrations of the compounds and on the $O_2$ status. Protection by sulphydryls is generally much greater in $O_2$ than in hypoxic conditions (see Alper, 1979, for review). Recently, however, the dependence of radioprotection on the $O_2$ concentration has been shown to be more complex (Lunec *et al.*, 1981; Denekamp *et al.*, 1981). The maximum radioprotection effect of dithioerythritol *in vitro* was obtained at 0·3% $O_2$, with less protection in anoxia *or* in air (Cullen *et al.*, 1980). Similarly, using the epidermal colony assay *in vivo*, the maximum effect of WR 2721 was seen in air, being reduced in 100% $O_2$ or in low $O_2$ concentrations (Denekamp *et al.*, 1981). Thus, both *in vitro* and *in vivo* the maximum radioprotection was obtained in the region of the "K" value* for $O_2$, *i.e.* in the region where small changes in the available $O_2$ have the most marked effect on the radiosensitivity of the system (Denekamp *et al.*, 1981, 1982). The early data of Dewey (1963) using very high concentrations of cysteine and high-pressure $O_2$ on *Serratia marcescens* accord with this conclusion.

Our data for MISO and WR 2721 also show a similar interaction. The radiation-modifying action of either compound could be reduced or eliminated by an appropriate dose of the other (Fig. 5 & 6, Table II). These data are also consistent with the hypothesis of redox competition for radical fixation of the initial chemical lesions that lead to biological damage. They confirm *in vivo* the basic mechanisms which have been elucidated for competition between sensitizers and protectors in the variety of *in vitro* studies mentioned above.

### *Clinical application*

Our data are more pessimistic in terms of the potential clinical usefulness of the drug combinations than earlier animal studies (Yuhas *et al.*, 1977; Sodicoff *et al.*, 1979; Grigsby & Maruyama, 1981). We have been unable to confirm their statements that there was independent action of the 2 drugs, whether in terms of toxicity, tumour sensitization or skin protection.

The drug doses used in all these experi-

* The "K" value is the $[O_2]$ at which half the maximum sensitization is obtained (Alper & Howard-Flanders, 1956).

ments are much higher than those likely to be tolerated in man. At present, the maximum tolerated dose clinically for MISO is 12 $gm/m^2$ in 6–30 fractions (Dische *et al.*, 1979) and for WR-2721 about 740 $mg/m^2$ for a single dose (Blumberg *et al.*, 1982). However, the interaction that we have found between these compounds occurs at all dose levels tested, and presumably similar but correspondingly smaller effects will pertain at clinically relevant dose levels.

Fig. 1 shows a very clear increase in WR-2721 toxicity if the animals are treated 15 min earlier with 200 or 670 mg/kg MISO. This is in marked contrast to the data of Yuhas *et al.* (1977) over the same MISO dose-range, but agrees well with the data of Grigsby & Maruyama (1981).

Figs. 5 & 6 summarize our experiments on the interaction of MISO and WR-2721 on the skin response to radiation. All 4 panels show a dose-dependent decrease in protection or sensitization when the opposing agent is added. These data are similar to the effect observed by Yuhas *et al.* (1977) for marrow, and are more marked than the slight interaction they reported for skin reactions on tumour-bearing limbs (Yuhas *et al.*, 1977). We have deliberately chosen to study normal-tissue responses in areas not compromised by a growing tumour, since the tumour growth may influence the skin reaction differently at different X-rays dose levels. We have allowed a longer time for WR-2721 penetration into the skin (30 min *vs.* 15 min) because earlier studies indicated that at least 30 min were needed to obtain maximum radioprotection (Stewart & Rojas, 1982; Travis *et al.*, 1982). It seems unlikely that this detail of timing could account for the greater magnitude of interaction in our studies, since the protection factors with 400 mg/kg WR-2721 alone are similar in our work and in Yuhas's (1·36–1·55 *vs.* 1·45–1·66). Furthermore the concept of competitive interaction is supported by the $O_2$ data since a 5-fold increase in inspired $O_2$ tension for 1–2 min before irradiation can also reduce the protection. Another report on the combined action of MISO and WR-2721 in normal tissues comes from Sodicoff *et al.* (1979). They demonstrated no reduction in the protection factor for rat salivary glands in the presence of 200 mg/kg MISO, but these experiments used historical controls for the rats treated with no drug or WR-2721 alone. Grigsby & Maruyama (1981) showed interaction of WR-2721 and MISO on the oral mucosa. They found a significant reduction in WR-2721 radioprotection with all the MISO combinations tested. Thus the interaction of MISO and WR-2721 has been demonstrated in 3 tissues *in vivo* (skin, marrow and oral mucosa).

Yuhas *et al.* (1977) also reported the effects of these 2 drugs separately and in combination on a tumour. They saw no interaction on the Line 1 carcinoma. By contrast we have observed a marked reduction in the MISO sensitization of 2 tumours (the fibrosarcoma SA FA and the anaplastic tumour CA MT) when the protector was added 30 min before irradiation (*i.e.* 15 min after the sensitizer). This interference with tumour sensitization was seen, even when no significant radioprotection by WR-2721 alone was obtained (Rojas *et al.* in preparation). These data indicate to us that competitive interaction can also occur in tumours, and that the radioprotector can diffuse and penetrate into the hypoxic cells (Rojas *et al.*, 1982). In both skin and tumours the balance of the competition between protection and sensitization depends upon the relative doses of the 2 drugs.

These experiments show that the combination of MISO and WR-2721 provides a powerful tool for indirect investigation of the $O_2$ tension in the critical cells of normal tissues. Previously, the lack of correlation between radioprotection and WR-2721 concentration in a tissue has been inexplicable. Denekamp *et al.* (1982) have proposed that small variations in intracellular $O_2$ tensions in different tissues may explain some of the wide variation in the protection factors that have been reported. For example, in the lung, which shows high drug concentrations, the high local $O_2$ tensions may compete effectively with the exogenous sulphydryls, reducing the overall protection. Other tissues could show little protection if their $O_2$ tension were below the critical range. The greatest protection would then be expected in tissues with intracellular $O_2$ tensions critically close to the oxygen "K" value. This hypothesis is obviously open to experimental verification by manipulation of the $O_2$ content in the inspired gas.

In summary, we are not optimistic about the clinical potential of sensitizers and protectors used in combination. The additive toxicity, the reduced skin radioprotection and the reduced tumour sensitization indicate that a therapeutic advantage is unlikely.

We wish to thank Professor J. F. Fowler for his encouragement and constructive criticism of our work and the Cancer Research Campaign for financial support.

### REFERENCES

ADAMS, G. E. (1978) Hypoxic cell sensitizers for radiotherapy. *Int. J. Radiat. Oncol. Biol. Phys.*, **4**, 135.

ALPER, T. (1979) Chemical Protection. In *Cellular Radiobiology*. Cambridge: University Press. p. 87.

ALPER, T. & HOWARD-FLANDERS, P. (1956) The role of oxygen in modifying the radiosensitivity of *E. coli B. Nature*, **178**, 978.

ASQUITH, J. C., FOSTER, J. L. & WILLSON, R. L. (1974) Metronidazole ("Flagyl"): a radiosensitizer of hypoxic cells. *Br. J. Radiol.*, **47**, 474.

BEGG, A. C., SHELDON, P. W. & FOSTER, J. L. (1974) Demonstration of hypoxic cell radiosensitization in solid tumours by metronidazole. *Br. J. Radiol.*, **47**, 399.

BLUMBERG, A. L., NELSON, D. F., GRAMKOWSKI, M. & 4 others (1982) Clinical trials of WR-2721 with radiation therapy. *Int. J. Radiat. Oncol. Biol. Phys.* (in press).

CHAPMAN, J. D., REUVERS, A. P., BORSA, J. & GREENSTOCK, C. L. (1973) Chemical radioprotection and radiosensitization of mammalian cells growing *in vitro*. *Radiat Res.*, **56**, 291.

CULLEN, B. H., MICHALOWSKI, A. & WALKER, H. C. (1980) Correlation between the radiobiological oxygen constant K, and the non-protein sulphydryl content of mammalian cells. *Int. J. Radiat. Biol.*, **38**, 525.

DENEKAMP, J. (1973) Changes in the rate of repopulation during multifraction irradiation of mouse skin. *Br. J. Radiol.*, **46**, 381.

DENEKAMP, J. & HARRIS, S. R. (1975) Tests of two electron affinic radio-sensitizers *in vivo* using regrowth of an experimental carcinoma. *Radiat. Res.*, **61**, 191.

DENEKAMP, J., MICHAEL, B. D., ROJAS, A. & STEWART, F. A. (1981) Thiol radioprotection *in vivo*: The critical role of tissue oxygen concentration. *Br. J. Radiol.*, **54**, 1112.

DENEKAMP, J., MICHAEL, B. D., ROJAS, A. & STEWART, F. A. (1982) Radio-protection of mouse skin by WR-2721. The critical influence of oxygen. *Int. J. Radiat. Oncol. Biol. Phys.*, **8** (in press).

DEWEY, D. L. (1963) The X-ray sensitivity of *Serratia marcescens*. *Radiat. Res.*, **19**, 64.

DISCHE, S., SAUNDERS, M. B., FLOCKHART, I. R., LEE, M. E. & ANDERSON, P. (1979) Misonidazole: A drug for trial in radiotherapy and oncology. *Int. J. Radiat. Oncol. Biol. Phys.*, **5**, 851.

DOUGLAS, B. G. & FOWLER, J. F. (1976) The effect of multiple small doses of X-rays on skin reactions in the mouse and a basic interpretation. *Radiat. Res.*, **66**, 401.

FOWLER, J. F., KRAGT, K., ELLIS, R. E., LINDOP, P. J. & BERRY, R. J. (1965) The effect of divided doses of 15 MeV electrons on the skin response of mice. *Int. J. Radiat. Biol.*, **9**, 241.

FOWLER, J. F. & DENEKAMP, J. (1979) A review of hypoxic cell radiosensitizers in experimental tumours. *Pharmacol. Ther.*, **7**, 413.

GRIGSBY, P. & MARUYAMA, Y. (1981) Modification of the oral radiation death syndrome with combined WR-2721 and misonidazole. *Br. J. Radiol.*, **54**, 969.

HALL, E. J., ASTOR, M., GEARD, C. & BIAGLOW, J. (1977) Cytoxicity of Ro-07-0582. Enhancement by hyperthermia and protection by cysteamine. *Br. J. Cancer*, **35**, 809.

HARRIS, J. W. & PHILLIPS, T. L. (1971) Radiobiological and biochemical studies of thiophosphate radioprotective compounds related to cysteine. *Radiat. Res.*, **46**, 362.

HENDRY, J. H. (1979) Quantitation of the radiotherapeutic importance of naturally-hypoxic normal tissues from collated experiments with rodents using single doses. *Int. J. Radiat. Oncol. Biol. Phys.*, **5**, 971.

KLIGERMAN, M. M., SHAW, M. T., SLAVIK, M. & YUHAS, J. M. (1980). Phase I clinical studies with WR-2721. *Cancer Clin Trials*, **3**, 217.

KOCK, C. J. & HOWELL, R. L. (1980) Combined radiation-protective and radiation-sensitizing agents. Cysteamine protects against cytotoxicity, radiosensitization and metabolism-enhanced radiosensitization of misonidazole against hypoxic cells. In *Radiation Sensitizers: Their Use in the Clinical Management of Cancer*. (Ed. Brady). New York: Masson. **Vol. 5**, 34, 239.

KOCH, C. J. & HOWELL, R. L. (1981) Combined radiation-protective and radiation-sensitizing agents. II. Radiosensitivity of hypoxic or aerobic Chinese hamster fibroblasts in the presence of cysteamine and misonidazole: implications for the "oxygen effect" *Radiat. Res.* **87**, 265.

LUNEC, J., CULLEN, B. M., WALKER, H. C. & HORNSEY, S. (1981) A cautionary note on the use of thiol compounds to protect normal tissues in radiotherapy. *Br. J. Radiol.*, **54**, 428.

MICHAEL, B. D. & HARROP, H. A. (1980) Time scale and mechanism of radiosensitization and radioprotection at the cellular level. In *Radiation Sensitizers: Their Use in the Clinical Management of cancer*. (Ed. Brady) New York: Masson. Vol. 5, 14.

PHILLIPS, T. L., KANE, L. & UTLEY, J. F. (1973) Radioprotection of tumour and normal tissues by thiophosphate compounds. *Cancer*, **32**, 528.

POTTEN, C. S. & HOWARD, A. (1969) Radiation depigmentation of mouse hair: The influence of local oxygen tension on radiosensitivity. *Radiat Res.*, **38**, 65.

REDPATH, J. L. & WILLSON, R. L. (1973) Reducing compounds in radioprotection and radiosensitization: Model experiments using ascorbic acid. *Int. J. Radiat. Biol.*, **23**, 51.

ROJAS, A., STEWART, F. A. & DENEKAMP, J. (1982) Experimental radiotherapy with WR-2721 and misonidazole. *Int. J. Radiat. Oncol. Biol. Phys.*, **8** (in press).

SODICOFF, M. CONGER, A. D., PRATT, N. E., SINESI, M. & TREPPER, P. (1979) Chemoprotection of the rat parotid gland by combined use of WR-2721 and Ro-07-0582. *Radiat. Res.* **80**, 348.

STEWART, F. A., DENEKAMP, J. & RANDHAWA, V. S. (1982) Radiosensitization of normal mouse skin by misonidazole: Single and fractionated dose studies. *Br. J. Cancer*, **45** (in press).

STEWART, F. A. & ROJAS, A. (1982) Radioprotection of mouse skin by WR-2721 in single and fractionated treatments. *Br. J. Radiol.*, **55**, 42.

TANAKA, Y. & SUGAHARA, T. (1980) Clinical experiences of chemical radiation protection in tumor radiotherapy in Japan. In *Radiation Sensitizers: Their Use in the Clinical Management of Cancer*. (Ed. Brady). New York: Masson. Vol. 5, 421.

TRAVIS, E. L., PAKIDAL, T. N., DELUCA, A. M. & FOWLER, J. F. (1982) The time course of radioprotection by WR-2721 in mouse skin. *Int. J. Radiat. Oncol. Biol. Phys.* (in press).

URTASUN, R. C., STURTHWIND, J., RABIN, H., BAND, P. R. & CHAPMAN, J. D. (1974) High dose metronidazole: A preliminary pharmacological study prior to its use as a radiosensitizer. *Br. J. Radiol.*, **47**, 297.

WASSERMAN, T. H., STETZ, J. & PHILLIPS, T. L. (1981) Clinical trials of misonidazole in the United States. *Cancer Clin. Trials*, **4**, 7.

WILLSON, R. L. & EMMERSON, P. (1970) Reaction of triacetoneamine-N-oxyl with radiation induced radicals from DNA and from deoxyribonucleotides in aqueous solution. In *Radiation Protection and Sensitization*. (Ed. Moroson & Quintiliani). London: Taylor & Francis. p. 73.

WITHERS, H. R. (1967) The effect of oxygen and anaesthesia on radiosensitivity *in vivo* of epithelial cells of mouse skin. *Br. J. Radiol.*, **40**, 335.

YUHAS, J. M. (1980) Active versus passive absorption kinetics as the basis for selective protection of normal tissues by S-2-(3-aminopropylamino) ethylphosphorothioic acid. *Cancer Res.*, **40**, 1519.

YUHAS, J. M. (1981) On the potential application of radioprotective drugs in radiotherapy. In *Radiation–Drug interactions in Cancer Management*. (Ed. Sokol). Wiley & Sons (in press).

YUHAS, J. M. & STORER, J. B. (1969) Differential chemoprotection of normal and malignant tissues. *J. Nat. Cancer Inst.*, **42**, 331.

YUHAS, J. M., YURCONIC, M., KLINGERMAN, M. M., WEST, G. & PETERSON, D. F. (1977) Combined use of radioprotective and radiosensitizing drugs in experimental radiotherapy. *Radiat. Res.*, **70**, 433.